# An integrated co-expression network analysis reveals novel genetic biomarkers for immune cell infiltration in chronic kidney disease

**DOI:** 10.3389/fimmu.2023.1129524

**Published:** 2023-02-17

**Authors:** Jia Xia, Yutong Hou, Anxiang Cai, Yingjie Xu, Wen Yang, Masha Huang, Shan Mou

**Affiliations:** ^1^ Department of Nephrology, Molecular Cell Lab for Kidney Disease, Ren Ji Hospital, Shanghai Jiao Tong University School of Medicine, Shanghai, China; ^2^ Department of Biochemistry and Molecular Cell Biology, Shanghai Key Laboratory for Tumor Microenvironment and Inflammation, Shanghai Jiao Tong University School of Medicine, Shanghai, China

**Keywords:** biomarkers, CKD, WGCNA, TCF21, DACH1, PBMC, DDX17

## Abstract

**Background:**

Chronic kidney disease (CKD) is characterized by persistent damage to kidney function or structure. Progression to end-stage leads to adverse effects on multiple systems. However, owing to its complex etiology and long-term cause, the molecular basis of CKD is not completely known.

**Methods:**

To dissect the potential important molecules during the progression, based on CKD databases from Gene Expression Omnibus, we used weighted gene co-expression network analysis (WGCNA) to identify the key genes in kidney tissues and peripheral blood mononuclear cells (PBMC). Correlation analysis of these genes with clinical relevance was evaluated based on Nephroseq. Combined with a validation cohort and receiver operating characteristic curve (ROC), we found the candidate biomarkers. The immune cell infiltration of these biomarkers was evaluated. The expression of these biomarkers was further detected in folic acid-induced nephropathy (FAN) murine model and immunohistochemical staining.

**Results:**

In total, eight genes (*CDCP1*, *CORO1C*, *DACH1*, *GSTA4*, *MAFB*, *TCF21*, *TGFBR3*, and *TGIF1*) in kidney tissue and six genes (*DDX17*, *KLF11*, *MAN1C1*, *POLR2K*, *ST14*, and *TRIM66*) in PBMC were screened from co-expression network. Correlation analysis of these genes with serum creatinine levels and estimated glomerular filtration rate from Nephroseq showed a well clinical relevance. Validation cohort and ROC identified *TCF21*, *DACH1* in kidney tissue and *DDX17* in PBMC as biomarkers for the progression of CKD. Immune cell infiltration analysis revealed that *DACH1* and *TCF21* were correlated with eosinophil, activated CD8 T cell, activated CD4 T cell, while the DDX17 was correlated with neutrophil, type-2 T helper cell, type-1 T helper cell, mast cell, etc. FAN murine model and immunohistochemical staining confirmed that these three molecules can be used as genetic biomarkers to distinguish CKD patients from healthy people. Moreover, the increase of TCF21 in kidney tubules might play important role in the CKD progression.

**Discussion:**

We identified three promising genetic biomarkers which could play important roles in the progression of CKD.

## Introduction

Chronic kidney disease (CKD) is a global public health issue with a prevalence of 13.4% (1). It is clinically defined as renal structure abnormalities or dysfunction (estimated glomerular filtration rate, eGFR < 60 ml/min/1.73m^2^) that has persisted for more than 3 months (2). CKD has a poor prognosis and can easily progress to end-stage renal disease (ESRD). Renal biopsy is an essential tool for diagnosing the pathology of CKD. Owing to the complex etiology and long-term cause, the molecular basis of CKD is not completely known, and it is difficult to predict patient responses to treatment (3, 4). With the development of omics technologies in the past decade, transcriptional bioinformatics analyses of chronic diseases have improved our understanding of molecular processes involved in CKD and have identified some novel biomarkers (5–7). In addition, integrated analyses using appropriate methods have identified some candidate genes based on the transcriptome data from such single-gene studies (8, 9). Kidney often suffers pathogenic immune responses against autoantigens in kidney or renal complication of systemic autoimmune diseases, which drive the renal disease, such as lupus nephropathy, membranous nephropathy and glomerulonephritis (10). The immune cell infiltration in kidney worsens the renal function and aggravates renal fibrosis (11). On the other hand, uremic retention solutes in blood have toxic effect on immune cells and contribute to systemic inflammatory and immune dysfunction (12). It is valuable to give new insight into the immune cell infiltration and find novel genetic biomarker for elucidating the molecular mechanism of CKD.

However, due to the invasion, frequent renal biopsy greatly increases the risk of complications. In end-stage CKD, many uremic toxins remain in the blood. Peripheral blood mononuclear cells (PBMC) respond to such environmental stimuli and undergo functional changes (13). Therefore, as a non-invasive method, blood tests can help identify key genes or pathways involved in CKD progression (14).

For bioinformatics analysis, the algorithm and dataset sample size have a major impact on analysis results. To our knowledge, no network analysis using multiple methods has been performed on CKD kidney tissue and PBMC samples to find key genes related with immune cell infiltration. Weighted gene co-expression network analysis (WGCNA) is an algorithm that can analyze gene expression profiles to filter out disease-related modules and find hub genes. It is the most commonly used algorithm when searching for genes associated with clinical characteristics (15). In this study, we aimed to use the WGCNA algorithm in combination with differentially expressed genes (DEGs) and least absolute shrinkage and selection operator (LASSO) analysis, integrating three Gene Expression Omnibus (GEO) databases comprising PBMC and kidney tissue samples, to obtain reliable key genes related with immune cell infiltration evaluation for distinguishing the CKD patients from healthy people. In addition, we used online kidney disease databases (www.nephroseq.org), immunohistochemical (IHC) staining detection and murine CKD model for verification.

## Results

### Flowchart of bioinformatics analysis

**Figure 1:**
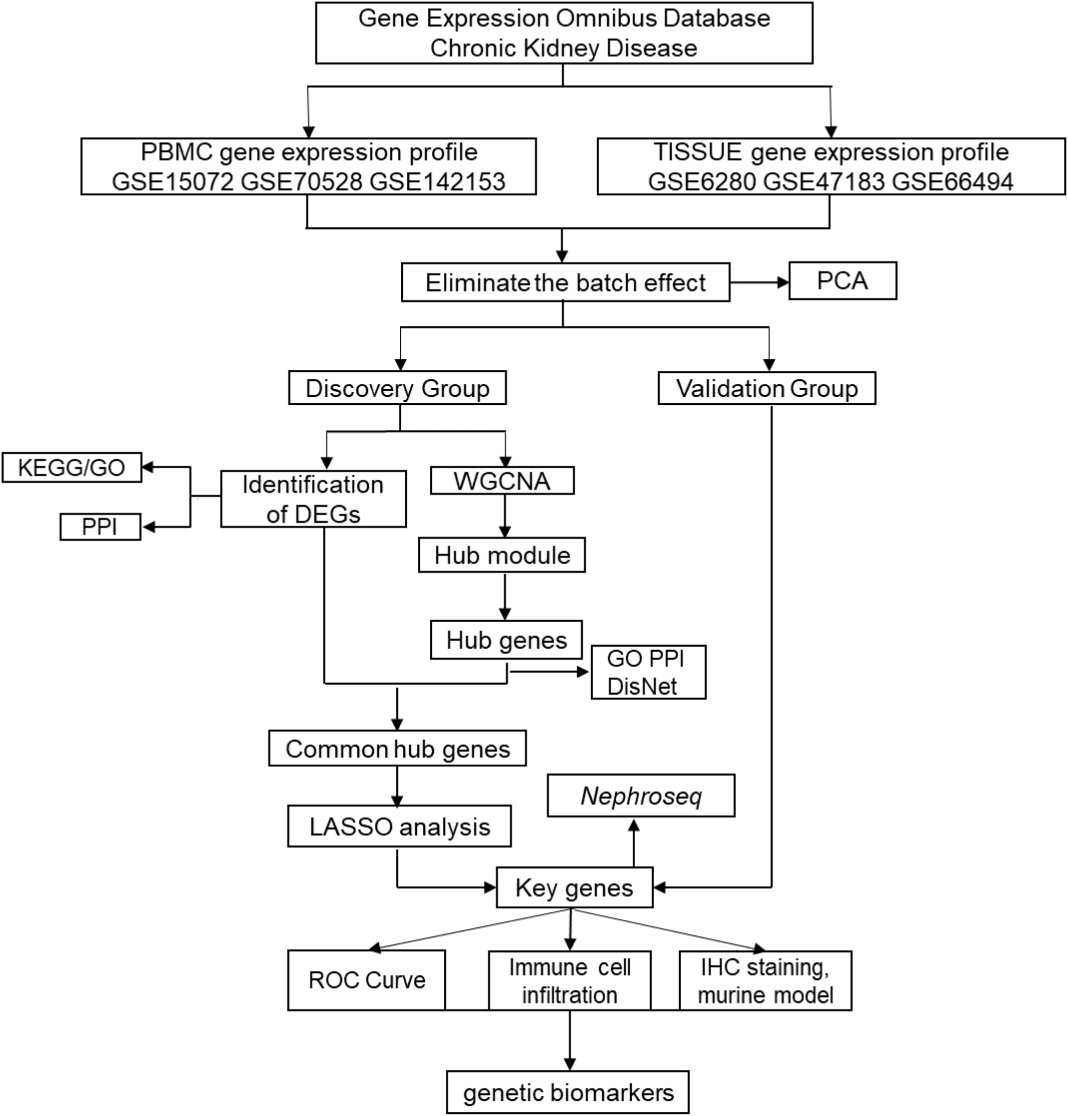
Flowchart to identify chronic kidney disease (CKD) biomarkers, including data extraction, processing and analysis.

The steps of our bioinformatics analysis are shown in the flowchart (**Figure 1**). Briefly, the CKD biomarker identification study was divided into the following main steps: (1) Peripheral blood mononuclear cell and renal tissue (TISSUE) data extraction and identification of DEGs; (2) Construction of co-expression network to identify hub genes; (3) Integrated analysis to extract key genes from DEGs and hub genes; (4) Expression validation to test credibility using validation group and external online cohort; (5) Multiple evaluation of candidate genes by online clinical database and murine experimental CKD model.

### Identification of DEGs in PBMC and renal tissue from CKD datasets

**Figure 2:**
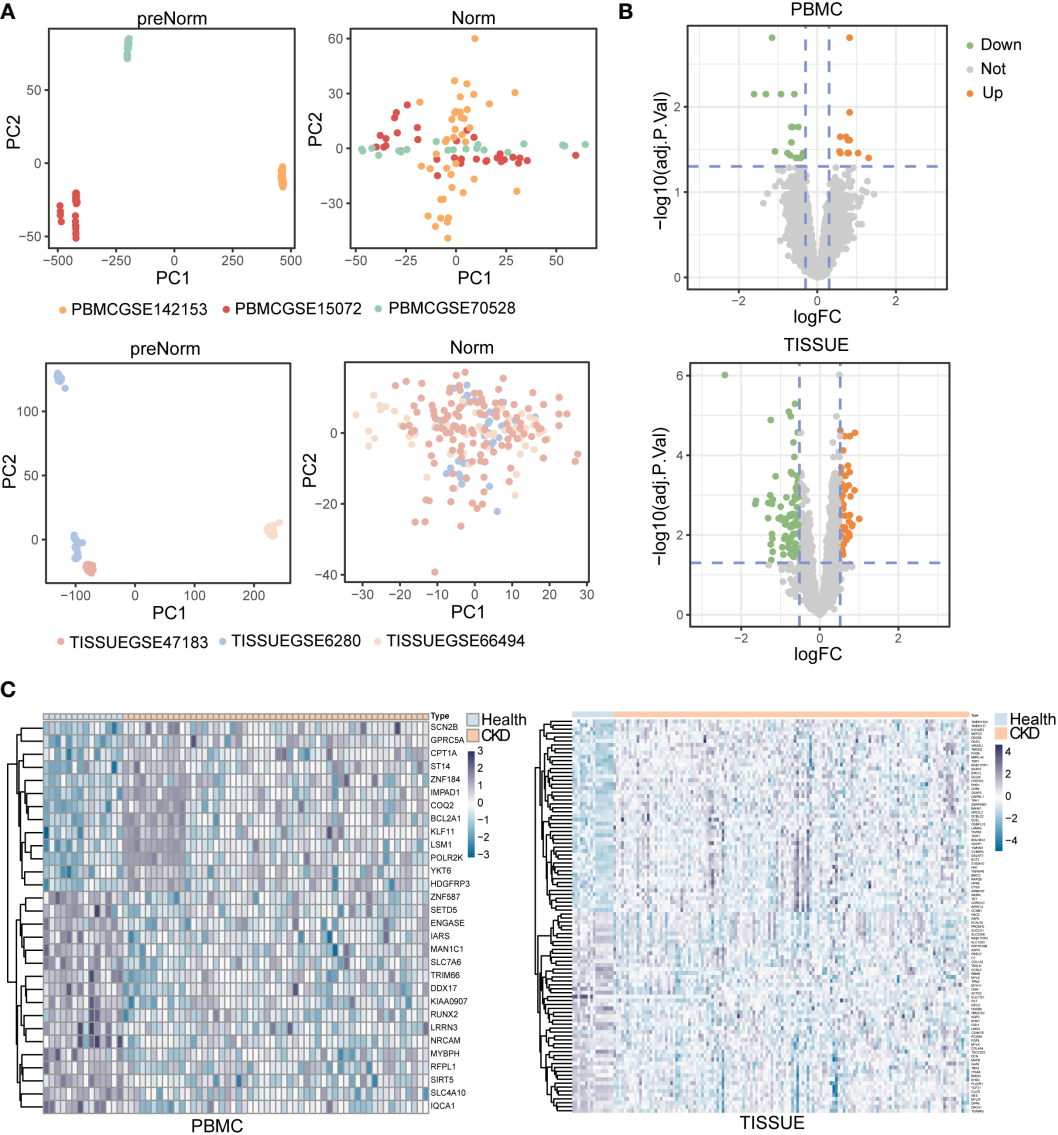
Identification of differentially expressed genes (DEGs) in peripheral blood mononuclear cell (PBMC) and TISSUE samples **(A)** Principal component analysis (PCA) of the GSM datasets. The samples were visualized by scatter plots based on two principal components (PC1 and PC2) of gene expression profiles without (left) or with (right) batch effect removal. Top, PBMC; bottom, TISSUE. **(B)** Volcano plots of the DEGs. The orange dots meant significantly upregulated genes, and the green dots represented significantly downregulated genes. The grey dots represented non-significantly changed genes. Top, PBMC; bottom, TISSUE. **(C)** Heatmap showing DEGs in different samples. Left PBMC, Right TISSUE.

First, we extracted transcriptome data from GEO datasets of CKD patients. In the clinic, blood tests and renal biopsies reveal various clinical features including abnormal kidney function, morphological changes and immunological dysfunction. In this study, GEO datasets of both PBMC and TISSUE samples were included and analyzed to explore the novel gene biomarkers in tissue and PBMC. The PBMC controls were from kidney-disease-free patients or healthy individuals and the TISSUE controls were from adjacent normal tissues of patients treated with tumor nephrectomy. To remove the batch effect, the R package ComBat was used. Principal component analysis (PCA) showed the normalized GEO samples (GSMs) from different GEO datasets (**Figure 2A**). GSMs were randomly divided into discovery and validation cohort (approximately a 4:1 ratio, **Table S1**). The DEGs had to meet the selection criteria (adjusted *P* < 0.05). Preliminarily, we found 30 DEGs from PBMC and 142 DEGs from TISSUE samples (**Table S2** and **S3**). **Figure 2B** shows the fold-change threshold (|log10(FC)| > 0.3 for PBMC and |Log10(FC)| > 0.52 for TISSUE), and **Figure 2C** showed the ranking of DEGs. Overall, we identified many DEGs in PBMC and kidney tissues from CKD patients, as compared with levels in controls. 

### Kyoto encyclopedia of genes and genomics and gene ontology analysis of DEGs

**Figure 3:**
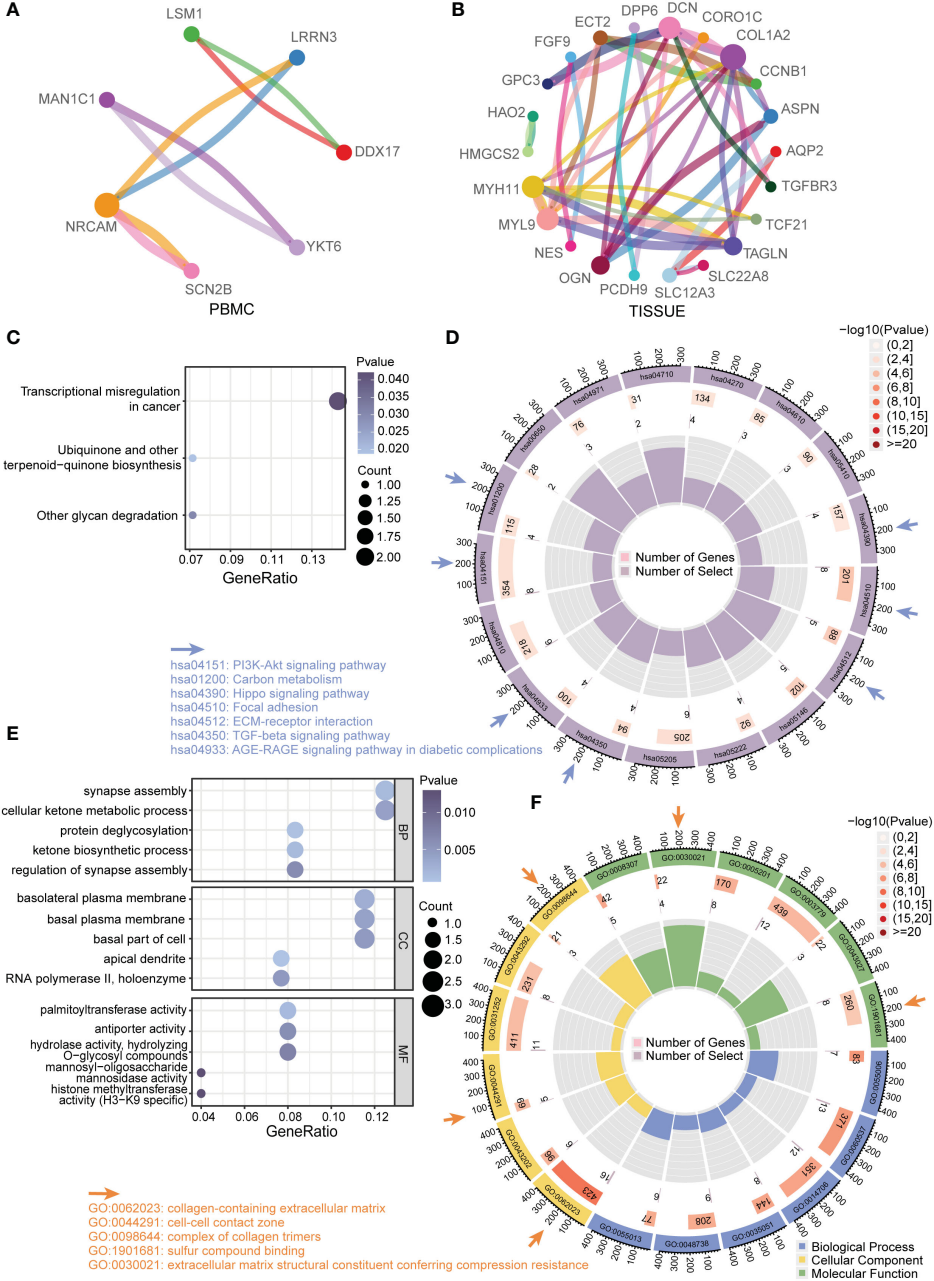
Functional enrichment analysis of DEGs **(A)** Protein–protein interaction (PPI) network of total DEGs from PBMC. Different colors of dots in the circle plot represented different proteins. The connectivity degree was represented by dot size. The edge width was proportional to combined score. **(B)** PPI network of total DEGs from TISSUE samples. **(C)** Enriched KEGG pathways among PBMC DEGs. The gene ratio was represented on the horizontal axis. The vertical axis indicated the KEGG signaling pathway terms, and the purple-to-blue gradually changing color indicated the change of significance from low to high. **(D)** Circular enrichment of KEGG pathways among TISSUE DEGs (hsa04510: Focal adhesion; hsa04512: ECM-receptor interaction; hsa05146: Amoebiasis; hsa05222: Small cell lung cancer; hsa05205: Proteoglycans in cancer; hsa04350: TGF-beta signaling pathway; hsa04933: AGE-RAGE signaling pathway in diabetic complications; hsa04810: Regulation of actin cytoskeleton; hsa04151: PI3K-Akt signaling pathway; hsa01200: Carbon metabolism; hsa00650: Butanoate metabolism; hsa04971: Gastric acid secretion; hsa04710: Circadian rhythm; hsa04270: Vascular smooth muscle contraction; hsa04610: Complement and coagulation cascades; hsa05410: Hypertrophic cardiomyopathy; hsa04390: Hippo signaling pathway). **(E)** Enriched GO terms among PBMC DEGs. **(F)** Circular enrichment of GO terms among TISSUE DEGs (GO:0055006: cardiac cell development; GO:0060537: muscle tissue development; GO:0014706: striated muscle tissue development; GO:0035051: cardiocyte differentiation; GO:0048738: cardiac muscle tissue development; GO:0055013: cardiac muscle cell development; GO:0062023: collagen-containing extracellular matrix; GO:0043202: lysosomal lumen; GO:0044291: cell-cell contact zone; GO:0031252: cell leading edge; GO:0043292: contractile fiber; GO:0098644: complex of collagen trimers; GO:0008307: structural constituent of muscle; GO:0030021: extracellular matrix structural constituent conferring compression resistance; GO:0005201: extracellular matrix structural constituent; GO:0003779:actin binding; GO:0043027: cysteine-type endopeptidase inhibitor activity involved in apoptotic process; GO:1901681: sulfur compound binding).

To interpret the general biological properties of DEGs, we used STRING to create the protein–protein-interaction (PPI) network. We found a close interaction among DEGs from PMBC (7/30) and TISSUE samples (22/142) (**Figures 3A, B**). Furthermore, we analyzed the DEGs *via* Kyoto Encyclopedia of Genes and Genomics (KEGG) pathway and Gene Ontology (GO) biological process analysis. The top KEGG terms associated with PBMC and TISSUE samples are shown in **Figures 3C, D**. PBMC DEGs were enriched in transcriptional misregulation in cancer, ubiquinone and other terpenoid-quinone biosynthesis and other glycan degradation, indicating the possible effect of the CKD status on energy metabolism and protein glycosylation. TISSUE DEGs were mainly involved in cell–ECM interactions, such as focal adhesion and ECM-receptor interaction. In addition, several signaling pathways were enriched, including TGF-beta signal pathway, AGE-RAGE signal pathway in diabetic complications, PI3K-Akt signal pathway and Hippo signaling pathway, which have been reported to play a role in CKD progression (16, 17). GO analysis results (**Figures 3E, F**) were similar to KEGG results. It can be seen that PBMC DEGs were involved in ketogenesis (cellular ketone metabolic process, ketone biosynthetic process), glycosylation processes (protein deglycosylation, hydrolase activity, hydrolyzing O-glycosyl compounds, etc.). In addition, TISSUE DEGs were enriched in ECM interactions and formation terms, such as collagen-containing extracellular matrix and complex of collagen trimers.

### WGCNA highlights and functional analysis of CKD-associated gene co-expression modules

**Figure 4:**
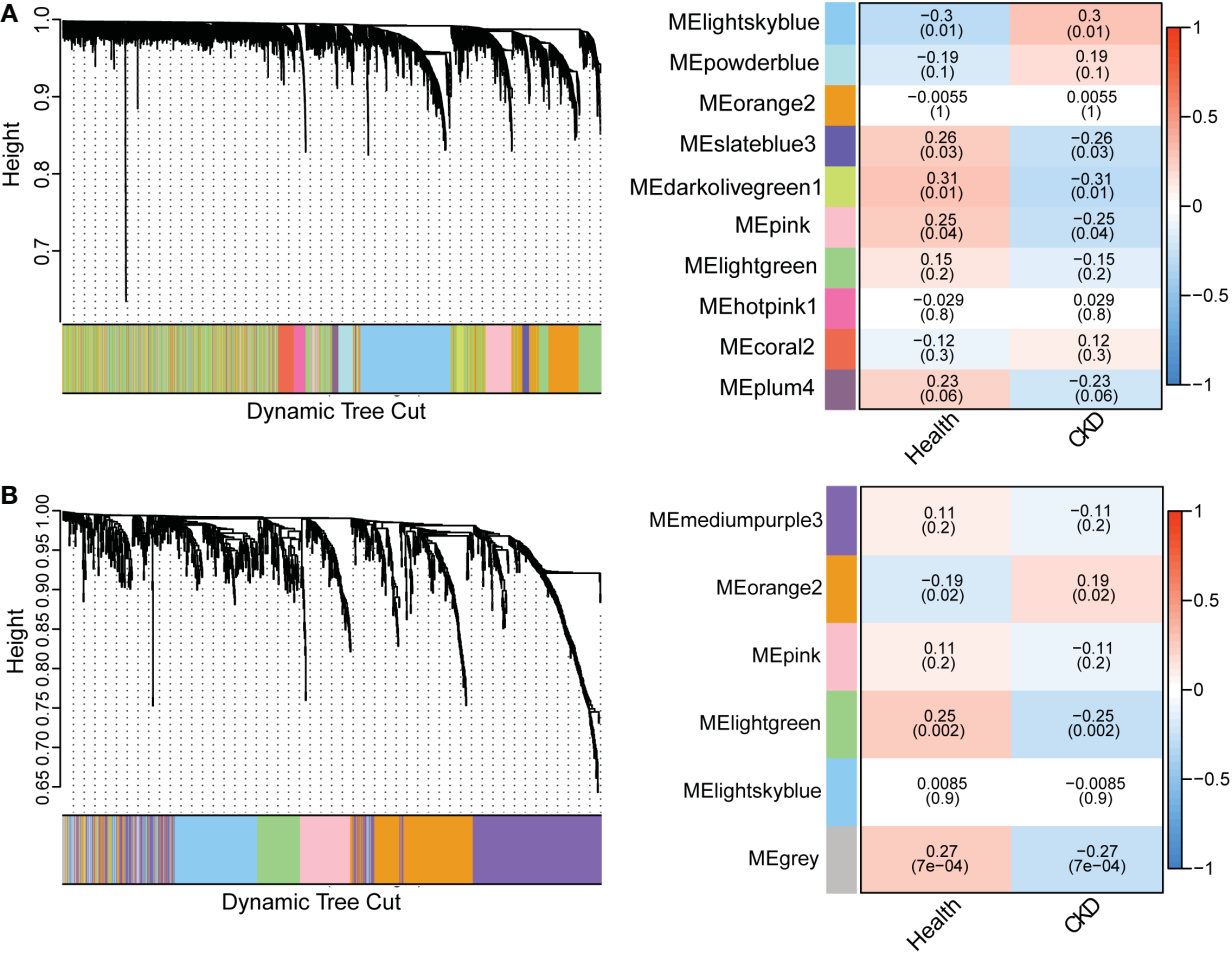
Weighted gene co-expression network analysis (WGCNA) revealing gene co-expression networks in samples from CKD patients **(A)** WGCNA analysis of PBMC samples. The left dendrogram represented the clusters of differentially expressed genes based on different metrics. Each branch represented one gene, and each color below branches represented one co-expression module. The right heatmap showed the correlation between gene modules and CKD. The correlation coefficient in each cube represented the correlation between gene modules and traits, which decreased from red to blue. **(B)** WGCNA analysis of TISSUE samples.

**Figure 5:**
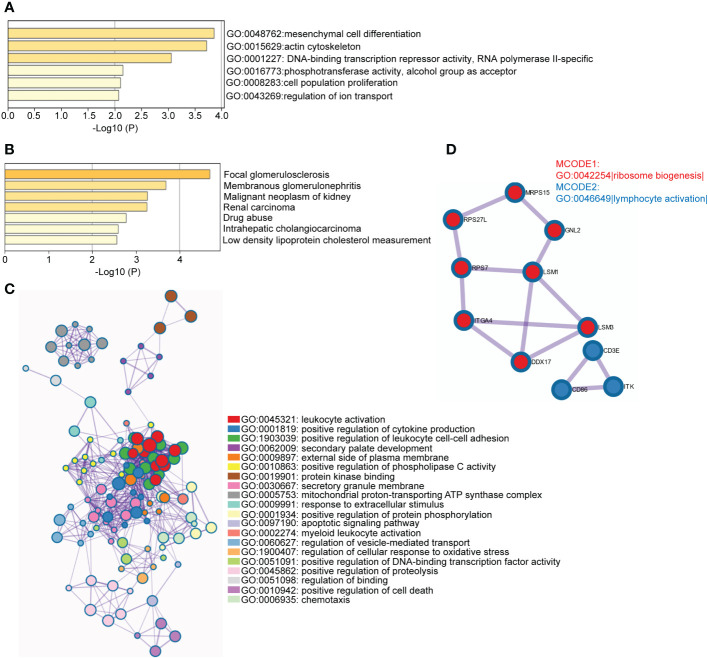
Functional enrichment analysis of hub genes in disease-related module **(A)** Enriched GO terms among TISSUE hub genes. The horizontal axis represented P-value of GO terms in log10 calculated on Metascape by default parameter. **(B)** Enriched DisGeNET terms among TISSUE hub genes. The horizontal axis represented P-value of GO terms in log10 calculated on Metascape. **(C)** Network of representative GO terms among PBMC hub genes. The clusters were calculated and visualized with Cytoscape using Metascape online platform by default parameter. The color of the node represented its cluster identity. One GO term from each cluster was selected to be shown as label. **(D)** Top MCODE terms of PBMC hub genes. All PPI among PBMC hub genes formed a network. The Molecular Complex Detection algorithm (MCODE) was used to identify the connected network components. The network was analyzed by GO enrichment to extract “biological meanings”. One GO term was labelled to represent the MCODE (GO: 0042254: Ribosome biogenesis; GO: 0046649: lymphocyte activation).

DEGs included only the most significantly regulated genes and other regulated genes may be missing from the transcripts. Here, we use WGCNA to robustly construct multiple gene co-expression modules. Hierarchical clustering was used to define branches of the cluster dendrogram in multiple randomly color-coded modules (**Figure 4A** left, PBMC; **Figure 4B**, left, TISSUE). The heatmap of correlations between module eigengenes and clinical traits (CKD or not) is shown in **Figure 4A** (right, PBMC) and **Figure 4B** (right, TISSUE). The darkolivegreen1 module (Corresponding Correlation, CC = −0.31, *P* = 0.01) showed the highest negative correlation with CKD trait in PBMC (**Figure 4A**, right). The lightgreen module (CC = −0.25, *P* = 0.002) showed a high negative correlation with CKD trait in TISSUE samples (**Figure 4B**, right). The hub genes of each sample type were filtered from these modules that met the selection module member (MM) and gene significance (GS) criteria described in the methods (**Table S4** and **S5**). Hub genes of the PBMC darkolivegreen1 module included *DDX17*, *KLF11*, *MAN1C1*, *POLR2K*, *ST14*, *TRIM66* and 121 other genes. Hub genes of the TISSUE lightgreen module include *CDCP1*, *CLIC5*, *CORO1C*, *DACH1*, *DENND2D*, *DPP6*, *GSTA4*, *MAFB*, *MAPK10*, *MYLIP*, *NEBL*, *NES*, *PDLIM2*, *PFKP*, *PLA2R1*, *TCF21*, *TGFBR3* and *GIF1*. The GO analysis of hub genes was performed on Metascape (http://metascape.org/). The top three GO terms of TISSUE hub genes were mesenchymal cell differentiation, actin cytoskeleton and DNA-binding transcription repressor activity, RNA polymerase II-specific (**Figure 5A**). DisNET analysis revealed that hub genes were closely related with glomerular disease (focal glomerulosclerosis and membranous glomerulonephritis) and renal carcinoma (**Figure 5B**). As for the hub genes of PBMC, the top three GO terms were leukocyte activation, positive regulation of cytokine production and positive regulation of leukocyte cell-cell adhesion (**Figure 5C**). Among them, The Molecular Complex Detection algorithm (MCODE) analysis enriched ribosome biogenesis and lymphocyte activation (**Figure 5D**). Above, the hub genes of TISSUE are closely correalted with DNA transcriptional dysregulation in kidney disease, while the hub genes of PBMC may actively affect the lymphocyte activation.

### Identification and validation of common hub genes

**Figure 6:**
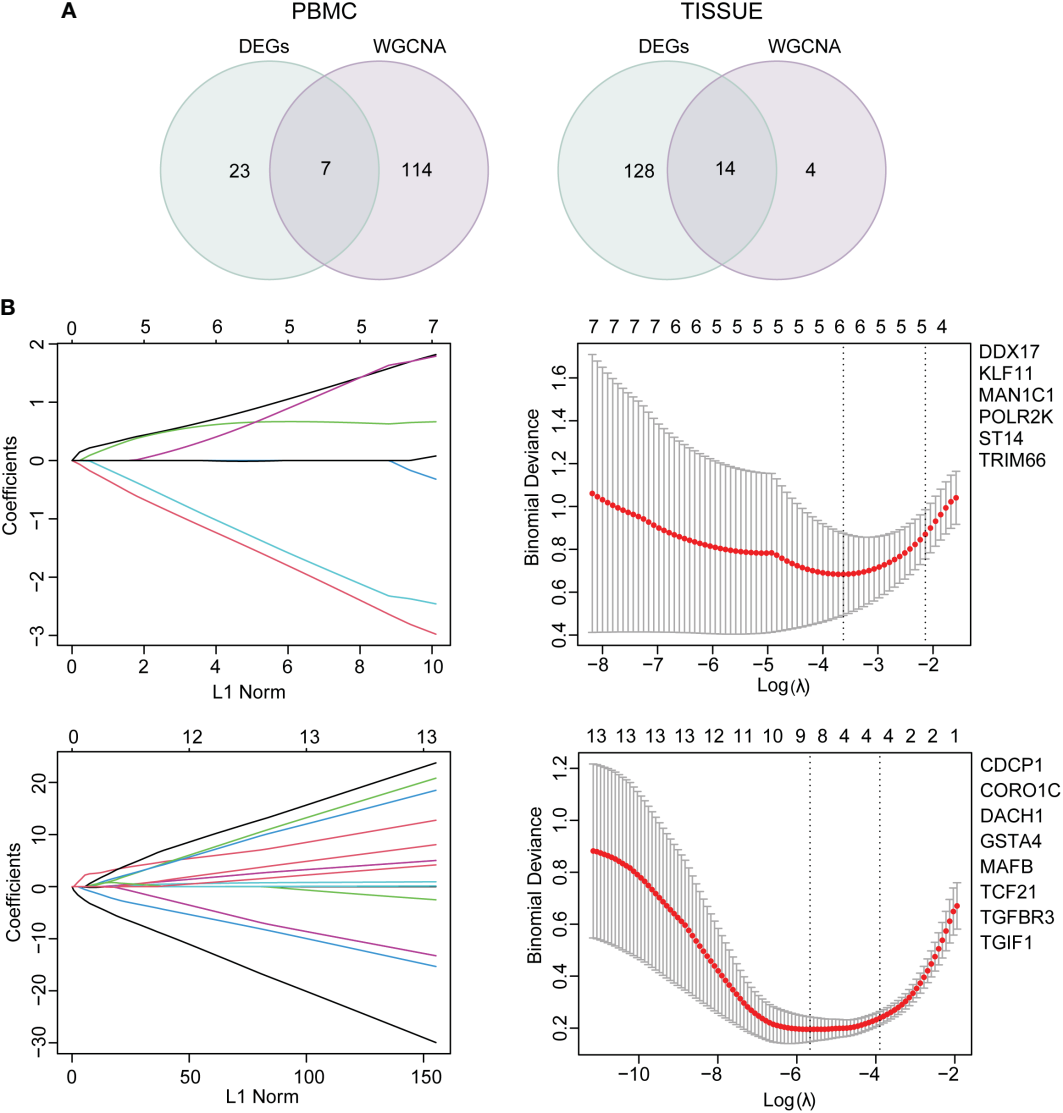
Common hub gene selection and least absolute shrinkage and selection operator (LASSO) analysis **(A)** The common hub genes shared between DEGs and hub genes were visualized in a Venn diagram. Left, PBMC; right, TISSUE. **(B)** The number of factors was determined by LASSO analysis. The procedure of LASSO Cox model fitting was shown in left panel. One curve represented a gene. The coefficient of each gene against the LC-norm was plotted with the lambda change. L1-norm represented the total absolute value of non-zero coefficients. A coefficient profile generated against the log (lambda) sequence was shown in the right panel. Continuous upright lines were the partial likelihood deviance ± SE; The optimal values and gene symbols were depicted, based on the minimum criteria (lambda.min, left vertical dotted line) and 1-SE criteria (lambda.1se, right vertical dotted line). Top, PBMC; bottom, TISSUE. SE, standard error.

**Figure 7:**
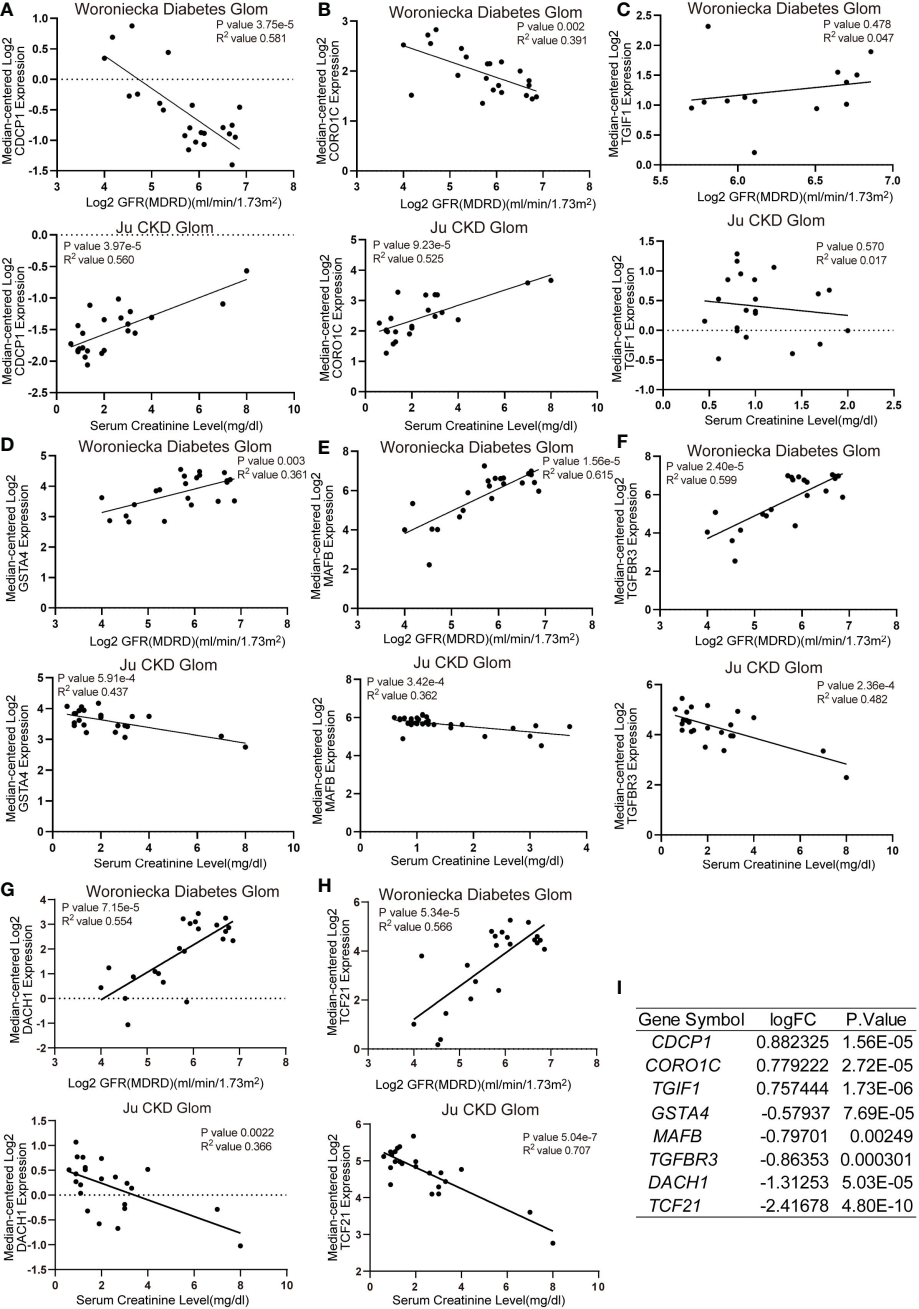
Correlation analysis of TISSUE common hub genes and CKD clinical parameters **(A–H)** The correlation of TISSUE genetic biomarker mRNA levels with estimated glomerular filtration rate (eGFR) in the Woroniecka Diabetes Glom Cohort (Top) or serum creatinine (SCr) level in the Ju CKD Glom Cohort (Bottom). **(A)** *CDCP1*; **(B)** *CORO1C*; **(C)** *TGIF1*; **(D)** *GSTA4*; **(E)** *MAFB*; **(F)** *TGFBR3*; **(G)** *DACH1*; **(H)** *TCF21*. Data were extracted from www.nephroseq.org. **(I)** The gene expression level of common hub genes in the discovery cohort.

Functional analysis based on hub genes did not exactly match the analysis based on DEGs. Therefore, to identify the key genes involved in CKD progression, we tried to define the common hub genes belonging to both DEGs and hub genes from WGCNA module (**Figure 6A**). 7 genes in PBMC and 14 genes in TISSUE were screened out. Using the LASSO regression algorithm, the common hub genes were reduced to six in PBMC (*DDX17*, *KLF11*, *MAN1C1*, *POLR2K*, *ST14* and *TRIM66*) and eight in TISSUE (*CDCP1*, *CORO1C*, *DACH1*, *GSTA4*, *MAFB*, *TCF21*, *TGFBR3* and *TGIF1*), which were identified as key genes (**Figure 6B**). Interestingly, *DDX17* and *MAN1C1* from PBMC hub genes and *CORO1C*, *TCF21* and *TGFBR3* from TISSUE hub genes were among the top-ranked genes in the PPI network. To examine whether the expression of these genes influenced CKD progression, we obtained clinical parameters (eGFR and serum creatinine, SCr) of these 8 tissue genes from www.nephroseq.org. Woroniecka Diabetes Glom database was used to evaluate the eGFR status, while the Ju CKD Glom was used to evaluate the SCr level. Consistently, the low-expressed genes, *GSTA4*, *MAFB*, *TGFBR3*, *DACH1* and *TCF21* expression was positively related with eGFR and negatively related with SCr. The high-expressed genes, *CDCP1*, *CORO1C* and *TGIF1* expression was negatively related with eGFR and positively related with SCr (**Figures 7A–H**). The expression and significance of these hub genes were highlighted in **Figure 7I**.

**Figure 8:**
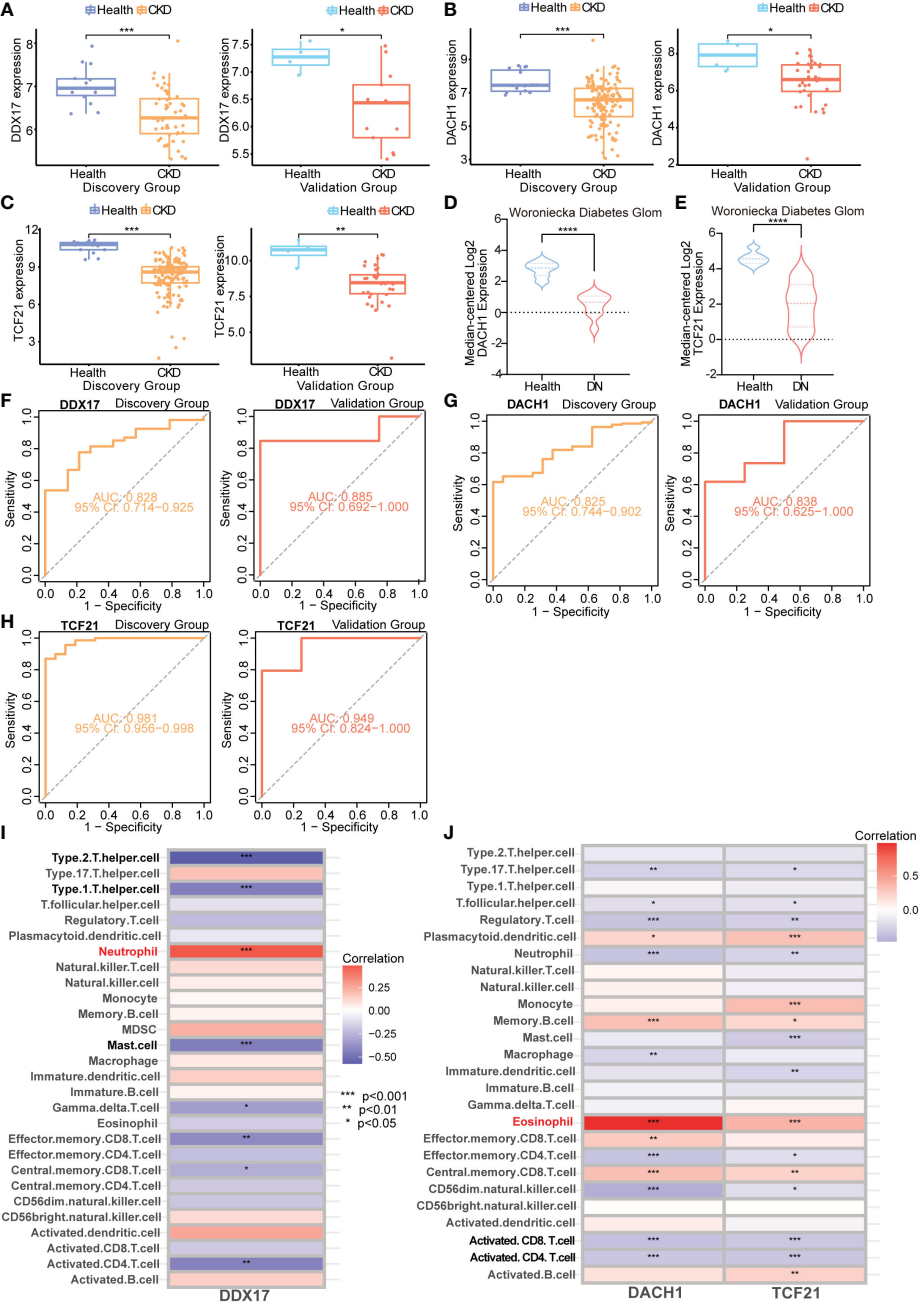
Validation and immune infiltration evaluation of CKD biomarkers from different samples **(A–C)** Box plots representing expression level of CKD genetic biomarkers in the discovery and validation cohorts. **(D)** mRNA level of *DACH1* in the Woroniecka Diabetes Glom Cohort. **(E)** mRNA level of *TCF21* in the Woroniecka Diabetes Glom Cohort. **(F–H)** Receiver operating characteristic (ROC) curve for the discovery and validation cohorts. **(A, F)** *DDX17* (PBMC); **(B, G)** *DACH1* (TISSUE); **(C, H)** *TCF21* (TISSUE). **(I)** Correlation heatmap demonstrating the relationship between *DDX17* (PBMC) and immune cells infiltration. **(J)** Correlation heatmap demonstrating the relationship between *DACH1* and *TCF21* (TISSUE) and immune cells infiltration. *P < 0.05; **P < 0.01; ***P < 0.001; ****P < 0.0001.

To determine the association between these genes and CKD status, we examined these 14 key genes in the validation cohort. Among them, the expression of *DDX17* in PBMC, *DACH1* and *TCF21* in TISSUE samples showed similar characteristics with those in the discovery cohort (**Figures 8A–C**). Consistently, *DACH1* and *TCF21* mRNA level were also decreased in the Woroniecka diabetic nephropathy cohort (**Figure 8D**, *DACH1*, *P* < 0.0001; **Figure 8E**, *TCF21*, *P* < 0.0001). Moreover, as shown in **Figures 8F–H**, receiver operating characteristic curve (ROC) was used to investigate whether these three key genes could discriminate between healthy and CKD samples. The classification accuracy (area under the ROC curve, AUC) of these three key genes (*DDX17*, *DACH1*, *TCF21*) was 0.828, 0.825 and 0.981 in the discovery cohort and 0.885, 0.838 and 0.949 in the validation cohort, respectively, showing strong ability to discriminate between CKD and healthy individuals. By reason of the foregoing, we screened out *DDX17*, *DACH1* and *TCF21* as genetic biomarkers. The correlation between parameters of renal function and the expression of genetic biomarkers suggested them may play a renoprotective role. Briefly, the renal *DACH1* and *TCF21* expression was positively correlated with eGFR in CKD patients (*DACH1*, *P* = 7.15e-5, R^2^ = 0.554; *TCF21*, *P* = 5.34e-5, R^2^ = 0.566), whereas it was negatively correlated with SCr levels (*DACH1*, *P* = 0.0022, R^2^ = 0.366; *TCF21*, *P* = 5.04e-7, R^2^ = 0.707).

### Immune cell infiltration analysis of genetic biomarkers

The TISSUE genetic biomarkers *DACH1* and *TCF21* are tumor suppressor genes. They are low expressed in the Kidney Renal Clear Cell Carcinoma (KIRC) (**Figures S1A, B**). The KIRC patients with high *DACH1* and *TCF21* expression has a better prognosis (**Figures S1C, D**). On the other hands, PBMC hub genes are closely related with lymphocyte activation (leukocyte activation, positive regulation of cytokine production, positive regulation of leukocyte cell-cell adhesion, etc.) (**Figure S1E**). The chronic kidney diseases and kidney malignant tumors have been demonstrated to be linked to a more severe immune cell infiltration. We evaluate the immune cell infiltration of these genetic biomarkers. Immune cell infiltration analysis revealed that *DDX17* in PBMC was found to be correlated with neutrophil, type 1 helper cell, type 2 helper cell and mast cell (**Figure 8I**). The TISSUE *DACH1* and *TCF21* were both found to be correlated with eosinophil, activated CD8 T cell and activated CD4 T cell (**Figure 8J**).

### CKD murine model and immunohistochemical validation of genetic biomarker expression

**Figure 9:**
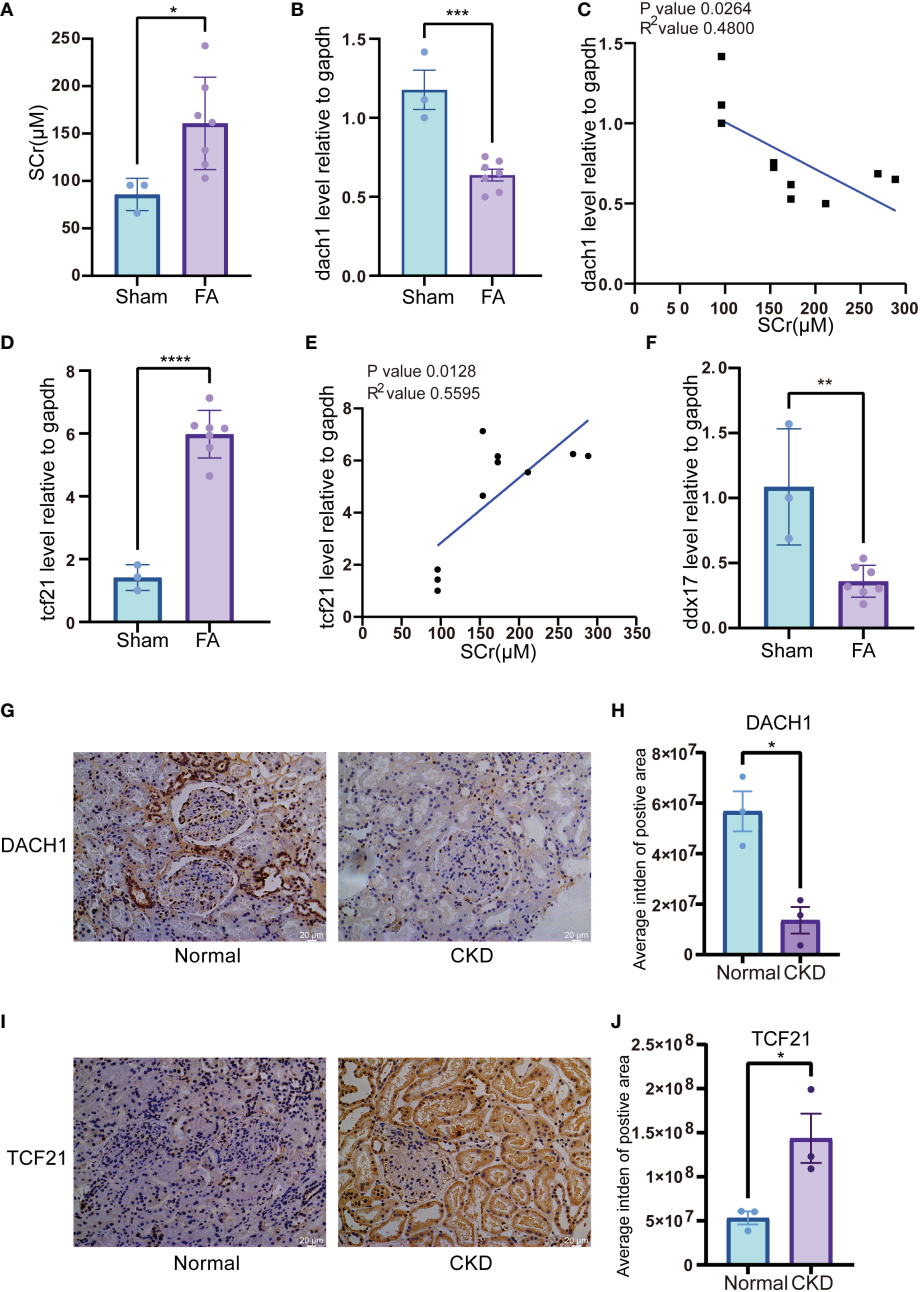
Expression of genetic biomarkers in folic acid (FA)-induced CKD murine model and membranous nephropathy (MN) patient’s biopsy **(A)** SCr level in FA-induced CKD murine model. Blood serum was harvested 3 days after FA injection. **(B)** mRNA level of *dach1* in kidneys from FA-induced CKD murine model. **(C)** Correlation between *dach1* mRNA level in kidney tissue and SCr from FA-induced CKD murine model. **(D)** mRNA level of *tcf21* in kidney tissues from FA-induced CKD murine model. **(E)** Correlation between *tcf21* mRNA level in kidney tissues and SCr from FA-induced CKD murine model. **(F)** mRNA level of *ddx17* in PBMC from FA-induced CKD murine model. **(G)** IHC staining of DACH1 in normal kidney tissues and CKD tissues. The representative pictures of CKD were from the membranous nephropathy (MN) patient’s biopsy. **(H)** Quantitative results of DACH1 expression in panel **(G)**. The expression level was calculated by average integrated density (Intden) of positive area. **(I)** IHC staining of TCF21 in normal kidney tissues and CKD tissues. **(J)** Quantitative results of TCF21 expression in **(I)**. *P < 0.05; **P < 0.01; ***P < 0.001; ****P < 0.0001.

To mimic renal dysfunction and tubulointerstitial fibrosis status during CKD, we developed a folic acid (FA)-induced CKD murine model. The results showed that SCr levels were increased significantly after a 3-day FA treatment (**Figure 9A**). At day 28, *dach1* mRNA levels in the kidneys of mice treated with FA were significantly decreased (**Figure 9B**, *P* < 0.001) and negatively correlated with SCr levels (**Figure 9C**, *P* = 0.0264, R^2^ = 0.4800). Conversely, *tcf21* mRNA levels were significantly increased (**Figure 9D**, *P* < 0.0001) and positively correlated with SCr levels (**Figure 9E**, *P* = 0.0128, R^2^ = 0.5595). In isolated PBMC, *ddx17* mRNA levels were significantly downregulated in the FA-treated kidney (**Figure 9F**, *P* < 0.01). These results showed that in the FA-induced CKD mouse model, *dach1* levels in tissue and *ddx17* levels in PBMC were in agreement with the changes in clinical samples. Interestingly, *tcf21* was significantly increased in the FA-induced CKD mouse model, opposite to the transcriptome change in clinical samples. Furtherly, IHC staining showed the protein expression of DACH1 and TCF21 in kidney of CKD patients and normal control. Both DACH1 and TCF21 were mainly expressed in the nucleus of glomerulus and renal tubule cells. DACH1 expression decreased in glomerulus and renal tubule of membranous nephropathy (MN) (**Figures 9G, H**, *P* < 0.05), while TCF21 expression increased in MN, especially in the renal tubule cells (**Figures 9I, J**, *P* < 0.05).

## Discussion

CKD is a type of kidney disease in which kidney function deteriorates and/or the structure is abnormal. Owing to its heterogeneity in etiology, the mechanism underlying its occurrence and progression is still not well understood. It is known that the TGF-β/Smad pathway is usually activated in CKD (18). However, damage to kidney function causes uremic retention solutes to remain in the body, resulting in adverse effects such as inflammation, immune dysfunction and oxidative stress (19–21), but the biological processes involved are not yet fully understood. In this study, we analyzed the gene expression profile of kidney tissues and PBMC from CKD patients, using various bioinformatics methods to find novel common genes or signaling pathways.

In the first-round screening, based on pathway enrichment analysis, signal molecules such as TGF-β, AGE-RAGE, PI3K-AKT and HIPPO play important roles in CKD progression and cross-talk with other molecules (16, 17, 22, 23). Based on GO analysis, the major functional terms are mainly enriched in cell adhesion and cell-extracellular matrix interactions, among others, which are involved in the critical pathological processes associated with CKD (24).

It is currently believed that WGCNA is better at identifying internal functional connections among regulated genes than DEG analysis. Wang et al. discovered that high expression levels of *CEBPZ*, *IFI16*, *LYAR*, *BRIX1*, *BMS1* and *DDX18* in the kidneys are potential key markers of CKD occurrence and progression (8). However, few studies combining WGCNA and clinical parameters using both kidney tissues and PBMC have been performed. In our study, 6 key genes (*KLF11*, *MAN1C1*, *POLR2K*, *ST14*, *TRIM66*, *DDX17*) in PBMC samples and 8 key genes (*CDCP1*, *CORO1C*, *GSTA4*, *MAFB*, *TGFBR3*, *TGIF1*, *TCF21* and *DACH1*) in TISSUE samples were identified by WGCNA. Several of these genes in tissue have been reported to contribute to the pathology and molecular changes in CKD. MAFB is a transcription factor that mediates renal tubule development and macrophage maturation (25). TGIF1 can bind to the MH1 domain of SMAD to inhibit TGF-β pathway activation (26). Most of them have a well correlation with SCr and eGFR in the CKD database. Among the PBMC common hub genes, several genes have been reported to be associated with kidney disease or hematologic disorder. KLF11 is a Krüppel-type zinc finger protein whose deficiency enhances chemokine generation and fibrosis in murine unilateral ureteral obstruction (27) and highly induced by TGF-β (28). It has been reported that KLF11 inhibits the activity of SMAD7 and enhances TGF-β pathway activation (29), through which KLF11 may influence the lymphocyte function. Besides, the highly upregulation of *ST14* promotes cancer cell invasion *via* imbalanced matriptase pericellular proteolysis (30, 31). The upregulation of *ST14* in PBMC may promote the inflammatory activation of endothelial cells in blood vessel, which is a common uremia-related complication. Thus, these molecules should be studied intensively. Finally, by examining the validation cohort and ROC curve, we identified *TCF21* and *DACH1* in TISSUE samples and *DDX17* in PBMC as potential biomarkers for CKD. The correlation of *DACH1* and *TCF21* with clinical parameters (eGFR and SCr) in CKD patients suggested that these molecules are potential renoprotective biomarkers in kidney tissues.

Dachshund family transcription factor 1 (DACH1) has been previously described as a tumor suppressor that can inhibit breast cancer invasion and metastasis (32). In the past several years, many studies have indicated that DACH1 is a renal-protective molecule. GWAS analysis showed that loss of DACH1 function was a susceptibility factor for renal fibrosis (33), and DACH1 can protect against podocyte damage in diabetic nephropathy model mice (34). Moreover, tubule-specific *DACH1*-knockout mice were more susceptible to renal damage and fibrosis in a FA-induced nephropathy model (33). In our study, this gene was identified through transcriptome bioinformatics analysis and confirmed based on clinical parameters, FA-induced nephropathy model and the IHC staining in kidney tissues of CKD patient. This agreement with reported studies showed that our bioinformatics analysis was reliable.

Transcription factor 21 (TCF21) is a transcription factor that plays an important role in the differentiation of mesenchymal cells and the development and maturation of gonads, muscle, kidney and other organs (35). *TCF21*-knockout mice developed kidney dysplasia at the embryonic stage and die after birth (36). Mice with podocyte-specific *TCF21*-knockout spontaneously developed proteinuria and exhibit FSGS (focal segmental glomerulosclerosis) lesions (37). Our bioinformatic analysis showed that *TCF21* mRNA levels were decreased in CKD samples and positively correlated with eGFR. However, in our FA-induced nephropathy murine model, *TCF21* mRNA levels were elevated compared to control group. The TCF21 staining signal was mainly in the nucleus in the healthy kidney, while in our mild case of membranous nephropathy, the signal increased and appeared in the cytoplasm and brush border of renal tubules. Such inconsistency may be caused by the following reasons. First, the protective role of TCF21 was mainly reported in the podocytes, not in the kidney tubules. This finding is supported by analyzing the tissue datasets of CKD cohort mainly from glomerular transcriptome (GSE47183 with 100 glomerular transcriptome samples and GSE66494 with 41 whole kidney transcriptome samples). Combining the two datasets may bias the display of key genes in the glomeruli during CKD progression. Recent study identified TCF21 as a deactivation factor of fibrogenic HSCs in liver fibrosis (38). It might be a nephroprotective in tubulointerstitial fibrosis. In our mouse model, high dose of folic acid mainly destroys the tubules and leads to the consequent tubulointerstitial fibrosis. Therefore, in the acute injury stage and early CKD stage of our mouse model, the upregulated TCF21 in tubule could play a protective role against kidney injury. In fact, TCF21 protein levels were also elevated in the early stage of diabetic nephropathy in model mice (39). Second, TCF21 was normally expressed in nuclei of podocytes and highly accumulate in both nuclei and cytoplasma of the injured podocytes in glomerular diseases, even was detected in urine (40). The severe injury of podocytes in CKD might lead to the loss of this cell population which lead to the reduction of total *TCF21* mRNA in kidney tissue. It is in line with our bioinformatics analysis and previous reports.

Among key genes from PBMC, we identified *DDX17*, which an ATP-dependent RNA helicase that is a coactivator of DNA-regulated transcription factors and is involved in mRNA transcription, splicing and maturation (41). *DDX17* is an immune-related gene defined in immunology database and analysis portal (ImmPort). In our study, it was expressed at low levels in the PBMC of CKD patients from the GEO database and in the PBMC of FA-induced CKD mouse model. In PBMC, DDX17 plays an important role in innate immunity against virus invasion (42–44). DDX17 is an essential mediator of sterile NLRC4 inflammasome activation (45). Given the fact that the uremia-associated immune deficiency is a well-known complication of CKD and it increase the risk of virus-infection and virus-associated cancers (46), the low DDX17 level in PBMC might associated with CKD progression.

As for the interrelationship of these three biomarkers. DDX5/DDX17 complex can co-activate or co-repress transcription factor transcription. In kidney tissue, the expression of *DDX17* was also decreased in the Woroniecka Diabetes Glom database, which was in line with the *DACH1* and *TCF21* expression. There is no report on the relationship between these three genes in the occurrence and development of CKD. Mechanically, we speculated that the low expression of DDX17 might further down-regulate the activity of TCF21 and DACH1, worsen the glomerular sclerosis and renal interstitial fibrosis. Given its critical role in innate immunity against virus invasion, the low DDX17 level in PBMC may aggravate immune deficiency in ESRD (42–45), and may result in chronic inflammation and increased oxidative stress to exacerbate kidney injury and loss of renal function (47).

During the course of CKD, in addition to the abnormal activation of some signaling pathways such as TGF-β/Smad and PI3K-Akt, some central molecules are also lacking. The low activity of some key molecules that regulate kidney differentiation and development might lead to dysfunction. DACH1 and TCF21 are both important transcription factors for kidney development and maturation. Some studies have demonstrated the association between their expression and CKD and illuminated their glomerular-specific roles in kidney disease (33, 34, 36, 37, 48), but the exact mechanisms underlying their dysregulation in CKD are still elusive. In our study, GO analysis of DEGs and hub genes highlighted DNA transcriptional activity in CKD kidney tissues. Besides, the evaluation of immune cell infiltration showed a positive correlation of *DACH1*, *TCF21* expression with eosinophil, activated CD8 T cell and activated CD4 T cell. This analysis suggested that low expression of *DACH1* and *TCF21* may lead to pathological dysregulation through aberrant immune cell infiltration. Folic acid induced experimental nephropathy models undergo progression from acute kidney injury (AKI) to CKD with initial damage to proximal tubules, significant alterations to these two transcription factors suggest their overall impact on glomeruli and tubules.

Our study also had some limitations. For example, the included database did not distinguish between the different pathological types of CKD to find pathology-specific biomarkers. In addition, we mainly analyzed the mRNA levels of genes, not the protein level. In addition, many renal proteins can be detected noninvasively in urine. In a subsequent study, we hope to detect the protein levels of key genes under investigation.

## Materials and methods

### Clinical samples

In this study, we adopt three samples from healthy adjacent kidney tissues of individuals who were performed tumor nephrectomy, whereas other three samples as CKD group whose tissues were taken from CKD patients’ biopsy. The pathologist confirmed these biopsy samples as membranous nephropathy (MN). The patients in this study consented under the ethics committee review of Renji Hospital affiliated to Shanghai Jiao Tong University, School of Medicine.

### Folic acid-induced nephropathy murine model

C57bl/6J mice were obtained from Shanghai Jiao Tong University, School of Medicine. All mice were maintained with a 12-hour light-dark cycle and given food and water ad libitum. Procedures were performed in accordance with guidelines approved by the ethical committee of animal experiments, Shanghai Jiao Tong University, School of Medicine. Folic acid was purchased from Sangon Biotech and dissolved in 300 mM NaHCO_3_. 12-week-old mice were intraperitoneally injected with FA (250 mg/kg) or NaHCO_3_. Mice were euthanized and sacrificed 28 days after FA injection. Whole blood was collected in White’s buffer (pH 6.4). Ficoll-Paque (GE healthcare) was used to separate the PBMC according to the manufacturer’s instructions. Kidneys were collected and stored at −80°C for further study.

### Microarray data collection from GEO database

We downloaded 6 gene expression datasets (GSE142153, GSE15072, GSE70528, GSE47183, GSE6280, GSE66494) from the GEO database (https://www.ncbi.nlm.nih.gov/geo/). According to labeling information in the platform, probes were converted into their corresponding gene symbols. The GSE142153 dataset contained 7 CKD PBMC samples and 10 control PBMC samples. The GSE15072 dataset contained 26 CKD PBMC samples and 8 control PBMC samples. The GSE70528 dataset contained 11 CKD PBMC samples. The GSE47183 dataset contained 122 CKD kidney samples. The GSE6280 dataset contained 12 control kidney samples. The GSE66494 dataset contained 53 CKD kidney samples and 8 control kidney samples. In our study, the discovery cohorts from the GSE142153, GSE15072 and GSE70528 datasets were used to build co-expression networks and identify the predominant genes in the CKD PBMC samples. The discovery cohorts from the GSE47183, GSE6280 and GSE66494 datasets were used to build co-expression networks and identify the main genes associated with CKD kidney samples. The assignment of each sample to discovery or validation cohorts was shown in **Table S1**. The procurement and application for all data were in accordance with the guidelines and principles of the GEO databases.

### Data preprocessing

For both sample types (PBMC and TISSUE), each dataset was combined into a data matrix. ComBat from the R package sva was used to account for batch effects (49, 50). Whether the batch effect was removed was evaluated by PCA. The prcomp function in R was used to perform PCA. Data visualization was performed using the R packages ggplot2 (https://ggplot2.tidyverse.org) and RColorBrewer (https://cran.r-project.org/web/packages/RColorBrewer/index.html) unless otherwise noted.

### DEGs identification

The R packages limma and edgeR were used to identify the DEGs between CKD and normal samples, respectively (51, 52). Genes with an adjusted *P* < 0.05 and |log_10_(FC)| > 0.3 were selected as DEGs in PBMC. Genes with an adjusted *P* < 0.05 and |log_10_(FC)| > 0.52 were selected as DEGs in TISSUE. The R package pheatmap was used to generate heatmaps (53).

### Protein–protein-interaction network construction

STRING database (http://string-db.org) was used to build the PPI network, where a combined score > 0.4 was considered statistically significant (54). The CytoHubba Cytoscape plugin was used to calculate the nodes using the connectivity degree method (55). We then used the netVisual_circle function in the R package CellChat to visualize the PPI network (56).

### Functional enrichment analysis

We used the R packages Clusterprofiler and org.Hs.eg.db for Kyoto Encyclopedia of Genes and Genomics (KEGG) and Gene Ontology (GO) analysis (57). KEGG pathways or GO function terms with *P* < 0.05 were considered statistically significant. The R package circlize was used to create circos plots (58).

### Weighted gene co-expression network analysis

The R package WGCNA was used to constructed the co-expression network based on discovery cohorts (15). To merge modules that might be similar, 0.25 was defined as the cut-off height threshold. The phenotypes (CKD) were inputted into the co-expression network and the parameters modulus characteristic gene (ME), MM and GS were calculated. ME represented the important part in the PCA of each gene module and MM represented the connection between modules and genes. Correlation coefficients ≥ 0.50 and *P*-values < 0.05 were considered indicative of key modules for PBMC. Correlation coefficients ≥ 0.60 and *P*-values < 0.05 were considered indicative of key modules for kidney tissue. In the modular-trait correlation analysis, genes with high hub modularity were considered as hub genes. Hub genes of PBMC met the absolute values of GS > 0.20 and MM > 0.50. Hub genes of kidney tissue met the absolute values of GS > 0.20 and MM > 0.60.

### Common hub gene selection and LASSO analysis

Common hub genes were defined as the overlap between DEGs and WGCNA hub genes. Venn diagrams were prepared using the R package venn. LASSO was a regression analysis algorithm that performs both gene selection and classification (59). To select hub genes which were credibly associated with CKD, a logistic LASSO regression model was constructed based on common hub genes by R package glmnet. 10-fold cross-validation was performed for tuning parameter selection, and the partial likelihood deviance met the minimum criteria.

### Evaluation of immune cell infiltration

A set of genes that mark each infiltrating immune cell type was obtained (60). The correlation between gene expressions and immune cells infiltration was calculated using Pearson correlation analysis.

### The Cancer Genome Atlas verification of genetic biomarkers in TISSUE

GEPIA (http://gepia.cancer-pku.cn/index.html) is a customizable functionalities website for interactive analysis and visualization based on The Cancer Genome Atlas database (61). To further verify the two biomarkers of CKD kidney tissue, the GEPIA web server was used to plot gene expression level box plots between kidney renal clear cell carcinoma (KRIC) and normal tissues in the TCGA database. The patient data were grouped according to the transcripts per million (TPM) value. Log2 (TPM+1) was used for log-scale, and four-way analysis of variance (ANOVA) was applied. Overall survival analyses of biomarkers of kidney tissue were also performed using GEPIA.

### Validation of CKD biomarkers

The R package ggpubr was used to generate the gene expression box plot for hub biomarkers. The R package pROC was used to plot the ROC curves (62). The AUC values were calculated to evaluate the sensitivity and specificity of model (63).

### Kidney function

Serum creatinine (SCr) levels were evaluated through colorimetric assays based on Jaffe’s reaction using deproteinized serum samples (Nanjing Jiancheng). Absorbance was measured at OD510 nm (BioTek) and analyzed accordingly.

### Immunohistochemistry staining

5 um slides cut from 4% paraformaldehyde fixed and paraffin embedded kidney tissues as were obtained from pathology department of Renji Hospital. All sections were de-paraffinized followed by heat-induced antigen retrieval on a heating block in Tris-EDTA buffer, PH = 9.0 for 15 min. Primary Rabbit DACH1 antibody (Proteintech) and Rabbit TCF21 antibody (Sigma Aldrich) was used in a final solution of 1:200 overnight at 4°C. Secondary antibody was applied for 30 min at 37°C, and the color was developed using a diaminobenzidine peroxidase substrate kit (Dako REAL™ EnVision™, DAKO). Sections were then counterstained with hematoxylin, dehydrated and mounted. The expression of DACH1 and TCF21 were imaged by Leica Microscope X20.

### Real-time PCR

Mouse kidney tissues were homogenized in Trizol reagent (TianGen). Total RNA was extracted and reverse transcribed into cDNA (HiScriptIII RT SuperMix, Vazyme). Real-time PCR was performed on LightCycler480 apparatus (Roche) using SYBR Green Mix (Yeasen). Mouse *gapdh* was used as internal control gene. The relative gene expression was analyzed using 2^−ΔΔCT^ method. Primers were listed: *tcf21*-F, cgctcacttaaggcagatcc; *tcf21*-R, gtcaccacttccttcaggtca; *dach1*-F, cctgggaaacccgtgtactc; *dach1*-R, agatccaccattttgcactcatt; *ddx17*-F, gatcgggatcgtgacaggga; *ddx17*-R, agtcagtcttgctacttctggat; *gapdh*-F, tggccttccgtgttcctac; *gapdh*-R, gagttgctgttgaagtcgca.

### Statistical analyses

Data were shown as the Mean ± SEM. A two-tailed independent student’s test was conducted to assess statistical significance. The significance was denoted as follows: **** *p* < 0.0001; *** *p* < 0.001; ** *p* < 0.01; * *p* < 0.05; N.S., not significant.

## Data availability statement

The original contributions presented in the study are included in the article/**Supplementary Material**. Further inquiries can be directed to the corresponding authors.

## Ethics statement

The studies involving human participants were reviewed and approved by Ethics Committee of Renji Hospital, School of Medicine, Shanghai Jiao Tong University. The patients/participants provided their written informed consent to participate in this study. The animal study was reviewed and approved by ethical committee of animal experiments, Shanghai Jiao Tong University, School of Medicine.

## Author contributions

Conceptualization, SM and MH. Methodology, MH. Software, YH. Validation, YH and JX. Formal analysis, JX, YH and YX. Resources, AC. Writing—original draft preparation, JX. Writing—review and editing, WY, YX and SM. Visualization, JX. Supervision, SM and MH. Project administration, SM and MH. Funding acquisition, SM. All authors contributed to the article and approved the submitted version.
