# Supplementary material for: An integrated co-expression network analysis reveals novel genetic biomarkers for immune cell infiltration in chronic kidney disease
Source: Front Immunol. 2023 Feb 17;14:1129524. doi: 10.3389/fimmu.2023.1129524 (PMC9981626; doi:10.3389/fimmu.2023.1129524)
Supplement: Supplementary file 1 [file DataSheet_1.docx]

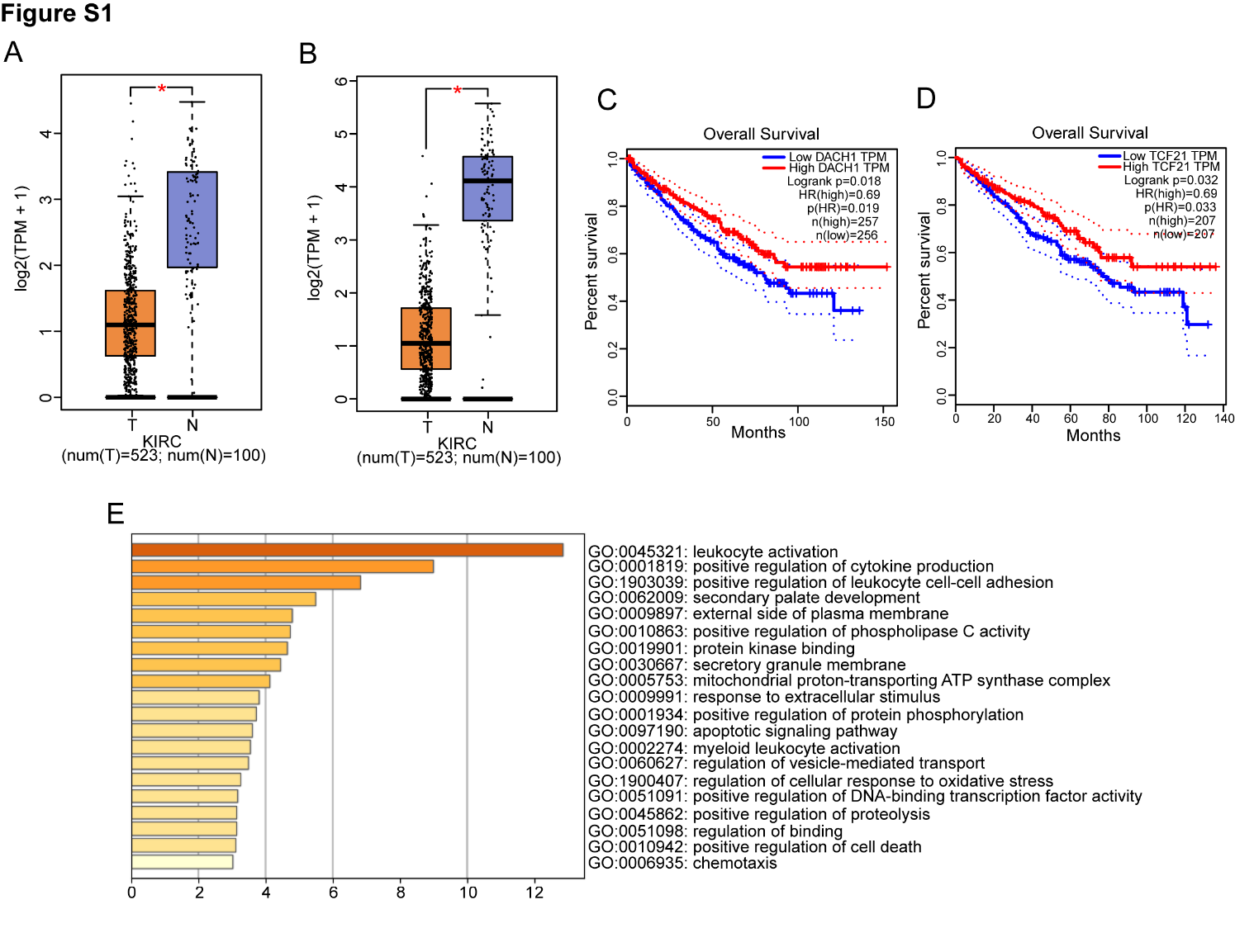


**Supplementary Figure 1**

**(A-B)** Box plot was derived from gene expression data in GEPIA comparing the expression of biomarkers in KIRC and normal tissues. **(A)** *DACH1*, **(B)** *TCF21*. **(C-D)** Overall survival analysis was performed using the GEPIA online platform. **(C)** *DACH1*, **(D)** *TCF21***. (E)** Enriched GO terms among PBMC hub genes. The horizontal axis represents P-value of GO terms in log base 10 calculated on Metascape by default parameter.
