# Supplementary material for: An integrated co-expression network analysis reveals novel genetic biomarkers for immune cell infiltration in chronic kidney disease
Source: Front Immunol. 2023 Feb 17;14:1129524. doi: 10.3389/fimmu.2023.1129524 (PMC9981626; doi:10.3389/fimmu.2023.1129524)
Supplement: Supplementary file 2 [file Table_1.docx]

## Table S1 Samples used for analysis.

A. PBMC

| Discovery group | | | Validation group | |
| --- | --- | --- | --- | --- |
| Health | CKD | | Health | CKD |
| GSM377067 | GSM4221602 | GSM377060 | GSM4221568 | GSM376036 |
| GSM4221572 | GSM4221589 | GSM375877 | GSM377069 | GSM4221595 |
| GSM4221575 | GSM375865 | GSM4221598 | GSM4221574 | GSM375881 |
| GSM4221571 | GSM4221582 | GSM4221590 | GSM377063 | GSM4221585 |
| GSM377066 | GSM375867 | GSM1808392 |  | GSM4221599 |
| GSM377064 | GSM4221579 | GSM4221597 |  | GSM4221593 |
| GSM4221576 | GSM4221601 | GSM375866 |  | GSM1808395 |
| GSM4221573 | GSM375868 | GSM375878 |  | GSM1808393 |
| GSM377065 | GSM4221596 | GSM377057 |  | GSM377061 |
| GSM377062 | GSM4221583 | GSM375872 |  | GSM1808394 |
| GSM4221570 | GSM375874 | GSM4221578 |  | GSM4221594 |
| GSM377068 | GSM4221584 | GSM375880 |  | GSM4221591 |
| GSM4221577 | GSM1808386 | GSM375862 |  | GSM4221581 |
| GSM4221569 | GSM375864 | GSM375863 |  |  |
|  | GSM4221587 | GSM377056 |  |  |
|  | GSM377059 | GSM375873 |  |  |
|  | GSM1808391 | GSM4221606 |  |  |
|  | GSM4221600 | GSM4221604 |  |  |
|  | GSM375875 | GSM1808389 |  |  |
|  | GSM1808387 | GSM375879 |  |  |
|  | GSM4221580 | GSM4221607 |  |  |
|  | GSM1808385 | GSM376040 |  |  |
|  | GSM4221586 | GSM1808390 |  |  |
|  | GSM4221603 | GSM1808388 |  |  |
|  | GSM376046 | GSM377058 |  |  |
|  | GSM4221588 | GSM375876 |  |  |
|  | GSM4221592 | GSM4221605 |  |  |

B. Kidney tissue.

| Discovery group | | | Validation group | |
| --- | --- | --- | --- | --- |
| Health | CKD | | Health | CKD |
| GSM144482 | GSM1146201 | GSM1623329 | GSM144474 | GSM1623340 |
| GSM144461 | GSM1146251 | GSM1623313 | GSM1623357 | GSM1146211 |
| GSM144483 | GSM1146224 | GSM1146320 | GSM144481 | GSM1146317 |
| GSM1623348 | GSM1623344 | GSM1623356 | GSM144493 | GSM1146277 |
| GSM144472 | GSM1623310 | GSM1146214 |  | GSM1146266 |
| GSM1623350 | GSM1146306 | GSM1146239 |  | GSM1146322 |
| GSM144494 | GSM1623338 | GSM1146221 |  | GSM1623326 |
| GSM144462 | GSM1146242 | GSM1146275 |  | GSM1146261 |
| GSM144492 | GSM1623331 | GSM1146282 |  | GSM1146308 |
| GSM1623349 | GSM1146260 | GSM1623343 |  | GSM1623353 |
| GSM144473 | GSM1146281 | GSM1146298 |  | GSM1623323 |
| GSM1623359 | GSM1146288 | GSM1146304 |  | GSM1146215 |
| GSM144463 | GSM1146241 | GSM1623324 |  | GSM1146253 |
| GSM1623358 | GSM1146290 | GSM1146249 |  | GSM1146254 |
| GSM1623351 | GSM1146252 | GSM1146223 |  | GSM1623330 |
| GSM1623347 | GSM1146225 | GSM1623317 |  | GSM1623309 |
|  | GSM1146300 | GSM1146268 |  | GSM1623315 |
|  | GSM1623332 | GSM1146271 |  | GSM1623352 |
|  | GSM1146243 | GSM1146246 |  | GSM1146202 |
|  | GSM1623304 | GSM1146295 |  | GSM1623341 |
|  | GSM1146274 | GSM1623318 |  | GSM1146314 |
|  | GSM1146316 | GSM1146226 |  | GSM1146294 |
|  | GSM1623328 | GSM1146289 |  | GSM1623333 |
|  | GSM1146276 | GSM1623335 |  | GSM1146206 |
|  | GSM1146311 | GSM1146216 |  | GSM1623336 |
|  | GSM1623334 | GSM1146321 |  | GSM1146203 |
|  | GSM1623308 | GSM1146259 |  | GSM1146237 |
|  | GSM1623346 | GSM1623301 |  | GSM1146305 |
|  | GSM1146273 | GSM1623345 |  | GSM1146210 |
|  | GSM1146257 | GSM1623337 |  | GSM1146231 |
|  | GSM1623322 | GSM1146270 |  | GSM1146208 |
|  | GSM1146296 | GSM1146284 |  | GSM1146272 |
|  | GSM1146250 | GSM1146227 |  | GSM1623299 |
|  | GSM1146263 | GSM1623342 |  | GSM1146318 |
|  | GSM1146232 | GSM1146299 |  |  |
|  | GSM1146248 | GSM1623320 |  |  |
|  | GSM1146283 | GSM1146217 |  |  |
|  | GSM1146238 | GSM1146222 |  |  |
|  | GSM1623303 | GSM1623305 |  |  |
|  | GSM1146204 | GSM1623321 |  |  |
|  | GSM1623311 | GSM1146209 |  |  |
|  | GSM1146240 | GSM1623316 |  |  |
|  | GSM1146236 | GSM1146235 |  |  |
|  | GSM1146265 | GSM1623319 |  |  |
|  | GSM1146207 | GSM1146233 |  |  |
|  | GSM1146315 | GSM1146301 |  |  |
|  | GSM1146267 | GSM1146245 |  |  |
|  | GSM1146247 | GSM1146278 |  |  |
|  | GSM1146230 | GSM1146302 |  |  |
|  | GSM1623306 | GSM1146280 |  |  |
|  | GSM1623300 | GSM1146205 |  |  |
|  | GSM1623354 | GSM1146293 |  |  |
|  | GSM1146220 | GSM1623327 |  |  |
|  | GSM1146287 | GSM1623307 |  |  |
|  | GSM1146255 | GSM1146313 |  |  |
|  | GSM1146228 | GSM1146244 |  |  |
|  | GSM1146319 | GSM1146307 |  |  |
|  | GSM1623314 | GSM1146285 |  |  |
|  | GSM1146219 | GSM1146258 |  |  |
|  | GSM1146279 | GSM1146218 |  |  |
|  | GSM1146291 | GSM1146309 |  |  |
|  | GSM1146312 | GSM1146297 |  |  |
|  | GSM1146229 | GSM1146234 |  |  |
|  | GSM1623325 | GSM1146256 |  |  |
|  | GSM1146303 | GSM1146212 |  |  |
|  | GSM1146292 | GSM1146262 |  |  |
|  | GSM1146269 | GSM1623339 |  |  |
|  | GSM1623312 | GSM1146264 |  |  |
|  | GSM1146286 | GSM1146213 |  |  |
|  | GSM1623302 | GSM1146310 |  |  |
|  | GSM1623355 |  |  |  |
